# A regulatory pathway model of neuropsychological disruption in Havana syndrome

**DOI:** 10.3389/fpsyt.2023.1180929

**Published:** 2023-10-27

**Authors:** Thomas P. Chacko, J. Tory Toole, Matthew C. Morris, Jeffrey Page, Robert D. Forsten, John P. Barrett, Matthew J. Reinhard, Ryan C. Brewster, Michelle E. Costanzo, Gordon Broderick

**Affiliations:** ^1^Center for Clinical Systems Biology, Rochester General Hospital, Rochester, NY, United States; ^2^War Related Illness and Injury Study Center (WRIISC), Department of Veterans Affairs, Washington, DC, United States; ^3^Department of Preventive Medicine and Biostatistics, Uniformed Services University, Bethesda, MD, United States; ^4^Complex Exposures Threats Center, Department of Veterans Affairs, Washington, DC, United States; ^5^Department of Medicine, Uniformed Services University, Bethesda, MD, United States

**Keywords:** neuropsychology, neuroimmunology, neurotransmission, mild traumatic brain injury, computational model, regulatory pathways, Havana syndrome, anomolous health incidents

## Abstract

**Introduction:**

In 2016 diplomatic personnel serving in Havana, Cuba, began reporting audible sensory phenomena paired with onset of complex and persistent neurological symptoms consistent with brain injury. The etiology of these Anomalous Health Incidents (AHI) and subsequent symptoms remains unknown. This report investigates putative exposure-symptom pathology by assembling a network model of published bio-behavioral pathways and assessing how dysregulation of such pathways might explain loss of function in these subjects using data available in the published literature. Given similarities in presentation with mild traumatic brain injury (mTBI), we used the latter as a clinically relevant means of evaluating if the neuropsychological profiles observed in Havana Syndrome Havana Syndrome might be explained at least in part by a dysregulation of neurotransmission, neuro-inflammation, or both.

**Method:**

Automated text-mining of >9,000 publications produced a network consisting of 273 documented regulatory interactions linking 29 neuro-chemical markers with 9 neuropsychological constructs from the Brief Mood Survey, PTSD Checklist, and the Frontal Systems Behavior Scale. Analysis of information flow through this network produced a set of regulatory rules reconciling to within a 6% departure known mechanistic pathways with neuropsychological profiles in *N* = 6 subjects.

**Results:**

Predicted expression of neuro-chemical markers that jointly satisfy documented pathways and observed symptom profiles display characteristically elevated IL-1B, IL-10, NGF, and norepinephrine levels in the context of depressed BDNF, GDNF, IGF1, and glutamate expression (FDR < 5%). Elevations in CRH and IL-6 were also predicted unanimously across all subjects. Furthermore, simulations of neurological regulatory dynamics reveal subjects do not appear to be “locked in” persistent illness but rather appear to be engaged in a slow recovery trajectory.

**Discussion:**

This computational analysis of measured neuropsychological symptoms in Havana-based diplomats proposes that these AHI symptoms may be supported in part by disruption of known neuroimmune and neurotransmission regulatory mechanisms also associated with mTBI.

## 1. Introduction

Several U.S. diplomatic personnel serving in Havana, Cuba, reported experiencing concussion-like symptoms from exposure to undetermined sources between 2016 and 2018 (1). The U.S. Department of State (DoS) has since finalized its implementation of the 2021 Helping American Victims Affected by Neurological Attacks (HAVANA) Act. This Act specifies that employees are eligible for compensation if they experienced an Anomalous Health Incident (AHI) that led to a traumatic brain injury (TBI), or TBI that required at least 12 months of medical treatment, or an acute onset of new debilitating persistent TBI symptoms, and the occurrence of the qualifying injury was on or after January 1, 2016. Though AHIs first emerged in 2016, with 40 U.S. embassy staff in Havana, Cuba they have since been reported by personnel in China and other overseas posts (2). The reported exposures were directional and multisensory in nature including auditory and haptic (vibrational) perceptions (3). Attributed to these AHI (4), popularly called Havana Syndrome (HS), many of the reported symptoms paralleled characteristic features of brain injury, prompting comparisons to mild traumatic brain injury (mTBI). More specifically, individuals who experienced these phenomena reported cognitive difficulties, including disorientation and memory loss, dizziness, nausea, headache, insomnia, fatigue, auditory symptoms including tinnitus, as well as vestibular and vision disturbances (5).

Driven by the possibility of an emerging threat, the DoS Office of Medical Services in 2017 requested that the Centers for Disease Control and Prevention (CDC) formulate a case definition for HS. The CDC reviewed medical records of individuals (*N* = 95) who were referred by DoS for evaluation or treatment at the University of Pennsylvania, the National Institutes of Health (NIH), and the University of Miami (UM), classifying the cases into (i) presumptive cases (*n* = 15), (ii) possible cases (*n* = 31), and (iii) not likely cases (*n* = 49) (6). The CDC proposed a biphasic symptom onset (primary and secondary symptom onset) criterion for both presumptive cases and possible cases, such that the initial onset of symptoms purportedly occurred while in Cuba or within 2 weeks of returning from Cuba. These cases included at least one of the following: head pressure, disorientation, nausea, headache, vestibular disturbances, auditory symptoms, and vision changes. Uniquely for presumptive cases with an unspecified timeframe, the secondary symptom onset included at least one of the following: vestibular disturbances and cognitive deficits. For possible cases the secondary symptoms or onset was not specified, instead, possible cases apparently included symptoms with an unknown onset, head pressure, disorientation, auditory symptoms, and vision changes. Of note, both the presumptive cases and possible cases were defined in the absence of alternative medical or other explanations for the reported symptoms.

Interestingly, most of the above symptoms also present in mTBI (5) even in the absence of any obvious physical blunt trauma. The typical mild-to-moderate HS symptoms are pervasive dysfunctions to cognitive, oculomotor, and central vestibular areas. These symptoms are also commonly seen in individuals with a history of mTBI or concussive injury (7). Tasked with the clinical evaluation, treatment, and rehabilitation of affected individuals referred to the University of Pennsylvania’s Center for Brain Injury and Repair by the US Department of State, Bureau of Medical Services, Swanson and colleagues (5) drew on these similarities and standard of care protocols typically applied to such clinical presentations only to find characteristic chronic cognitive, vestibular, and oculomotor disturbances in 21 of the individuals referred with an absence of head trauma history (5). These findings are consistent with early and albeit still limited evidence suggesting possible overlaps between HS and mTBI reported across concurrent neuroimaging studies, neuropsychological and biometric assessments as well as self-reports (5, 8, 9). Given these caveats, this seminal work by Swanson and colleagues was understandably met with strong skepticism. Two major criticisms included methodologically incorrect diagnostic classifications (10) and an alternative explanation suggesting that symptoms emerged secondary to a mass psychogenic disorder (11, 12). Given the emergent nature of the case definitions being proposed for HS and the complex and still evolving case definitions and guidelines (13) around mTBI, our use of this data in our work has focused on mapping basic neuroinflammatory and neurotransmission regulatory mechanisms to changes in symptom burden rather than in assignment to a specific illness subject group.

Despite intense debate regarding the etiology of Havana syndrome there remains a broad consensus acknowledging the chronic negative health outcomes of the phenomena and experience. Participants in an August 2021 meeting of The Joint Intelligence Community Council chaired by the Director of National Intelligence unanimously agreed to support National Security Council (NSC)-led interagency efforts to address AHI and expressed their view that identifying the cause of AHI is a top priority, as is providing the highest level of care to those affected, and supporting those affected by AHI to ensure they are believed, heard, and respected (14). This sentiment was also reflected in legislation. The National Defense Authorization Act (NDAA) for Fiscal Year 2022, Section 732, requires the Department of Defense (DoD) to provide medical assessment and individual treatment of personnel and their family members affected by AHIs. The Intelligence Authorization Act for Fiscal Year 2022 requires NSC to develop standardized protocols including: post-AHI medical testing of covered employees, covered individuals, and the dependents of covered employees; and protocols for baseline medical testing of covered US government personnel and their family members. As noted above, President Biden signed into law the HAVANA Act on October 8, 2021 (15). This act requires US government agencies to develop a process whereby employees of those agencies who are affected by AHIs, may apply for and receive a one-time, direct compensation payment from the agency.

In this work, while not ruling out any of the aforementioned plausible explanations for the reported symptoms and experiences, we examine symptom profiles reported by subjects investigated for HS through the lens of our aggregate knowledge of well-documented neurotransmission and neuroinflammatory pathways. We applied broad-scale automated text mining to extract and assemble bio-behavioral pathways into a regulatory network model, and then test in numerical simulations to assess if known neurological signaling could legitimately reproduce the altered neuropsychological profiles and loss of function reported in a small set of affected subjects suspected of suffering from HS. Preliminary results from these simulations suggest that such symptom profiles may indeed involve a dysregulation of many of the same neuropsychological and neuroinflammatory pathways also reported in association with mTBI. A subsequent text-mining directed at the recovery of specific statements in the Elsevier corpus describing the modulation of the neurologic pathway markers predicted to be differentially expressed by a given exposure source pointed to the involvement of pulsed electromagnetic radiation as one potential insult of interest, a finding consistent with a hypothesis recently posited by Nelson (3).

## 2. Methods

To leverage the limited amount of publicly available data describing this comparatively small number of subjects we applied a hypothesis driven approach. Rather than construct a model *de novo* from experimental data we draw on our prior knowledge of neurological signaling pathways and tested the resulting network predictions to determine if they adequately explained the clinical observations available.

### 2.1. Regulatory network assembly

**Figure 1:**
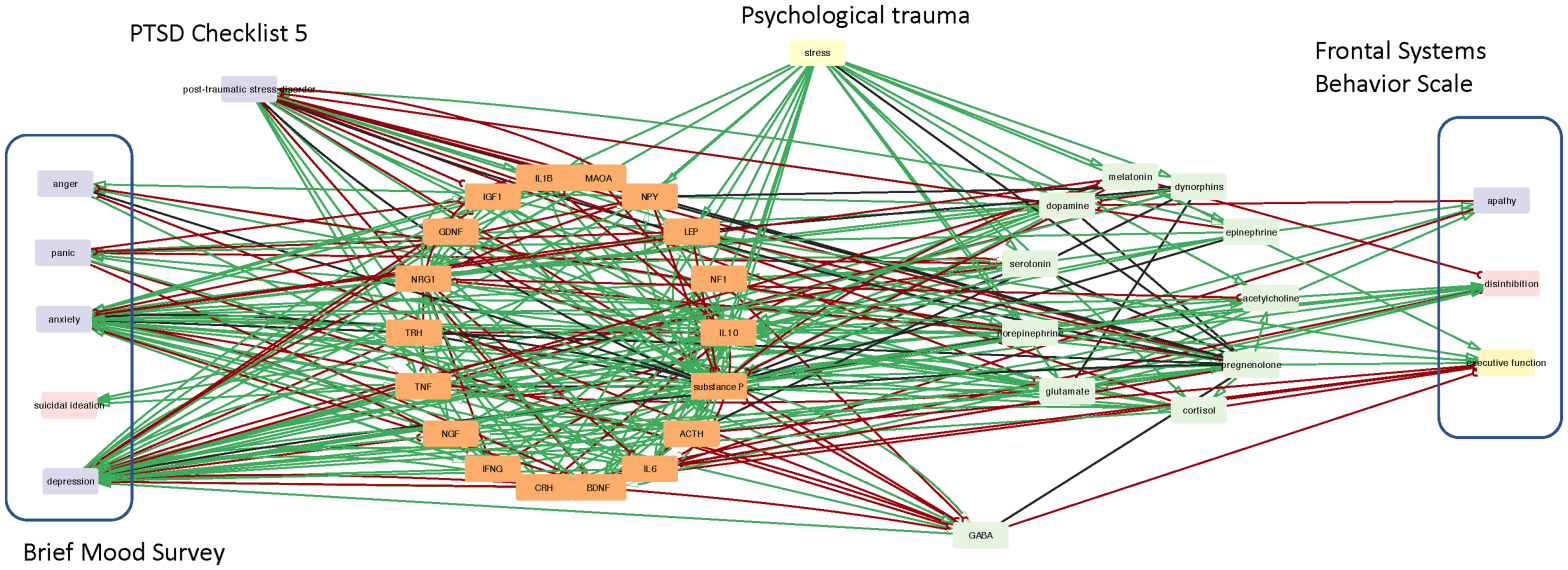
A text-mined bio-behavioral regulatory network. A network of documented neurotransmission and neuro-inflammation pathways associated with mTBI consisting of 29 molecular mediators, 9 mood function measures and an external stressor connected through 273 directed regulatory interactions supported 9,032 peer-reviewed citations. A green interaction indicates that a source node upregulates a downstream target. Conversely a red interaction indicates that a downstream target is down-regulated by its upstream mediator.

In this work we build on a bio-behavioral feedback network previously reported by our group to capture neurologic mechanisms relevant to mTBI (16), and further developed to explore co-morbidities in post-traumatic stress disorder (PTSD) (17). We extended this network again, incorporating in this iteration additional mediators involved in oculomotor control (18), as well as balance and vestibular function (19, 20), as disruptions to these systems have been reported in subjects suspected of suffering an AHI (5) and sharing clinical presentation broadly labeled as HS. More specifically, the putative regulatory circuit involved 29 molecular mediators such as brain-derived neurotrophic factor (BDNF), glial cell-derived neurotrophic factor (GDNF), insulin-like growth factor (IGF)-1, nerve growth factor (NGF), interleukin (IL)-1beta, interleukin-10 (IL-10), norepinephrine, glutamate and others. In addition to these neurologic markers the network includes neuropsychological function as described by 9 constructs reported by Swanson et al. (5) that include apathy, disinhibition, and executive functioning (frontal system behavior scale) along with anger, panic, anxiety, suicidal ideation, and depression (Brief mood survey). A node representing exogenous stress was included to capture response to psychological stressors. These 39 markers of brain signaling and behavioral function were linked through 273 regulatory interactions documented in 9,032 peer-reviewed publications (Figure 1) (Supplementary Tables S1, S2). The network was assembled iteratively with the first set of markers extracted from the Elsevier ontology being those markers reported to interact with at least one of 9 the neuropsychological measures. Markers in this first layer of molecular mediators were then linked to each other, often by recruiting additional intermediate markers, to form the augmented network. Finally, directed searches were conducted to identify intermediate mediators and relationships to ensure that all molecular mediators in the network were part of a closed regulatory feedback loop. In other words, no isolated source or terminal sink nodes were allowed.

It should be noted that in this first attempt only the Elsevier ontology and the MedScan reader were used. While not necessarily producing an exhaustive list of mediators, the basic assumption is that, given the extensive interdependencies linking neurotransmission and immune markers, the actions of those makers absent from the model are captured indirectly with sufficient fidelity. Testing model predictions against available observations will either validate or invalidate this assumption. In the latter case, a gap analysis will serve to determine around which network elements additional regulators should be considered.

The majority of these regulatory interactions were retrieved from the Elsevier Biology Knowledge Graph database (Elsevier, Amsterdam) (21) using the Pathway Studio interface (22). This database is updated weekly, and recognizes in excess of 1.4 M (million) biological entities (molecules, cell types, diseases, and clinical measures, etc…) connected through over 13.5 M relationships (co-expression, regulatory, and binding interactions, etc…) extracted by deploying the MedScan natural language processing (NLP) engine (23, 24) to over 5 M full-text peer-reviewed publications and over 32 M PubMed abstracts describing *in vitro* as well as *in vivo* animal and human studies (including results from over 300,000 clinical trials). In specific cases, the MedScan engine was applied using the Elsevier Text Mining (ETM) software suite to conduct highly focused semantic text mining to extract specific functional relationships not yet archived in the Knowledge Graph database, and/or to capture terms not yet included in the standard ontology. This was performed to assess and extract published reports linking specific electromagnetic exposures to characteristic changes in biomarker expression profiles predicted by the model.

### 2.2. Describing network structure

As a general indicator of network complexity, we computed the network connection density, or the total number of edges in the current network represented as a fraction of all the possible edges in a fully connected network with the same number of nodes. This measure is known to vary significantly across levels of biology and physiological compartments (25). In addition, as connection patterns in biological networks tend to favor the emergence of highly connected subnetworks, we also computed the network clustering coefficient (26, 27). At the level of component nodes, we computed different centrality measures to describe their relative role within the network (Supplementary Table S2). In addition to the number of upstream mediators (indegree) and downstream targets (outdegree) supported by each node, we also computed the closeness centrality to describe how well-connected the node is overall to the remainder of the network. This is computed as the average length of the shortest path between a given node and all other nodes in the network. To describe how a node might act as a key broker of information or gatekeeper between adjacent highly connected sub-networks, we computed the betweenness centrality. The measure is proportional to the frequency with which a node is positioned along the shortest paths between two other nodes. Finally, this same concept is extended to a describe the average shortest path length from a specific node to all other nodes in the network as an indication of how proximal it is to other nodes, and how directly information is transferred downstream. These network analyses were conducted using Cytoscape version 3.9.1^1^ (28).

### 2.3. A decisional logic model

**Figure 2:**
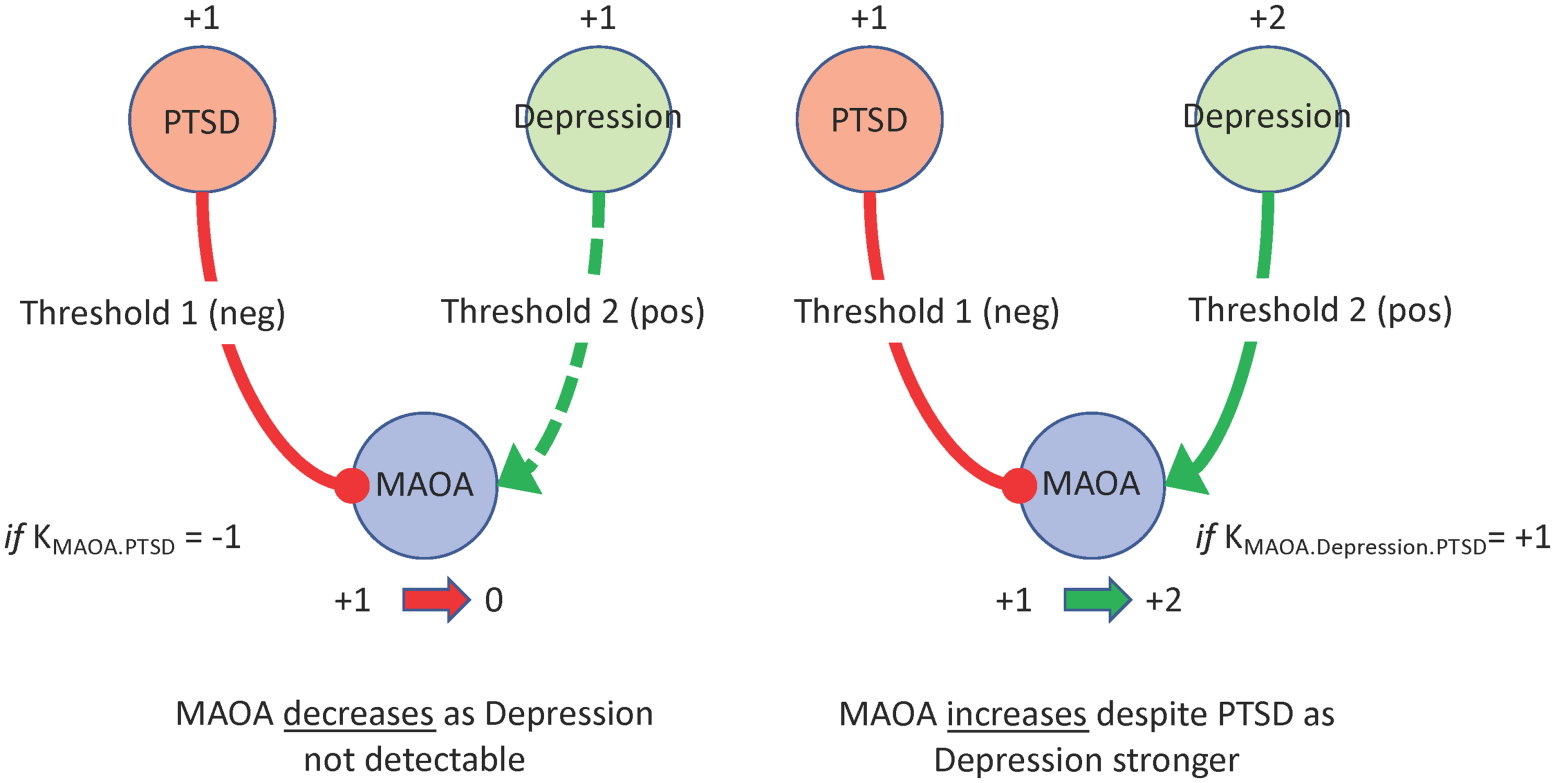
A regulatory logic program. In this network MAOA is part of a negative feedback through PTSD severity and a positive feedback through Depression severity. A putative regulatory program where Depression is only detectable by MAOA at high severity (+2) while PTSD is more readily detectable at moderate severity (+1), would result in a PTSD driven decrease in MAOA expression (left panel). If Depression increased to high severity (+2) it would become detectable making MAOA subject to the opposing actions of both upstream nodes. In this example a regulatory program might assign more influence to Depression over PTSD thereby driving an increase in MAOA (right panel).

Extending a formalism originally proposed by Thomas (29) and further developed by Mendoza and Xenarios (30), that described the regulatory dynamics of biological networks, we applied a discrete decisional logic to direct the flow of information through the behavioral feedback network and predict how the component markers and constructs change in expression across time (31). Each network regulatory interaction has a direction, i.e., a source and a target, as well as a mode of action whereby it will inactivate or activate a downstream target. The expression level or the extent to which each neurologic or behavioral node is activated is described in the current work as one of four discrete qualitative states, namely Low (0), Moderate (1), Severe/ High (2), and Very Severe/ Very High (3) (Supplementary Table S3). An increase or decrease in the activation level of any given node is determined by the states and actions of its upstream neighbors. The competing actions of upstream neighbors activated to levels above their respective perception thresholds are managed by a decision logic that weighs the actions of weak inactivators against strong activators, and vice versa, in a context-specific process before deciding to increase or decrease the activation of the node in question in the next iteration (31) (Figure 2). Sets of parameter values defining these decisional kinetics (logic weights and perception thresholds) were identified exhaustively by defining a computationally efficient constraint satisfaction problem (CSP) (32) where combinations of decisional logic parameter values were retained if they supported predicted dynamic responses that included behaviors observed experimentally (33, 34). These observed reference behaviors may be defined as transient or as stable persistent pathologies in which case the network is expected to not only accurately predict a specific neuropsychological profile, but also predict that it will remain unchanged in the next logical transition.

The degree to which a given regulatory network supported a plausible mechanistic explanation of HS was computed as the Manhattan distance separating observed neuropsychological profiles from those predicted by the model. Expressed as a fraction of the maximum possible misalignment, this overall unexplained departure was further decomposed into deviations at the level of individual network nodes (i.e., neurologic and behavioral markers) and individual subjects. The average predicted expression levels in HS subjects for each of the unobserved neurological markers was tested for significance using a one-sample *t*-test against similar predictions for an artificially-defined target control. The latter was defined as an idealized subject with a minimal severity profile and optimal function. The significance of these predicted differences was corrected for Type I errors from multiple comparison using a Benjamini-Hochberg procedure (35).

Identification of data-adherent parameter values, as well as all statistical analyses of model predictions, were conducted using a computational framework and tools developed by our team under Python version 3.8.3 (2020-05-13).^2^

### 2.4. Observed functional profiles

**Figure 3:**
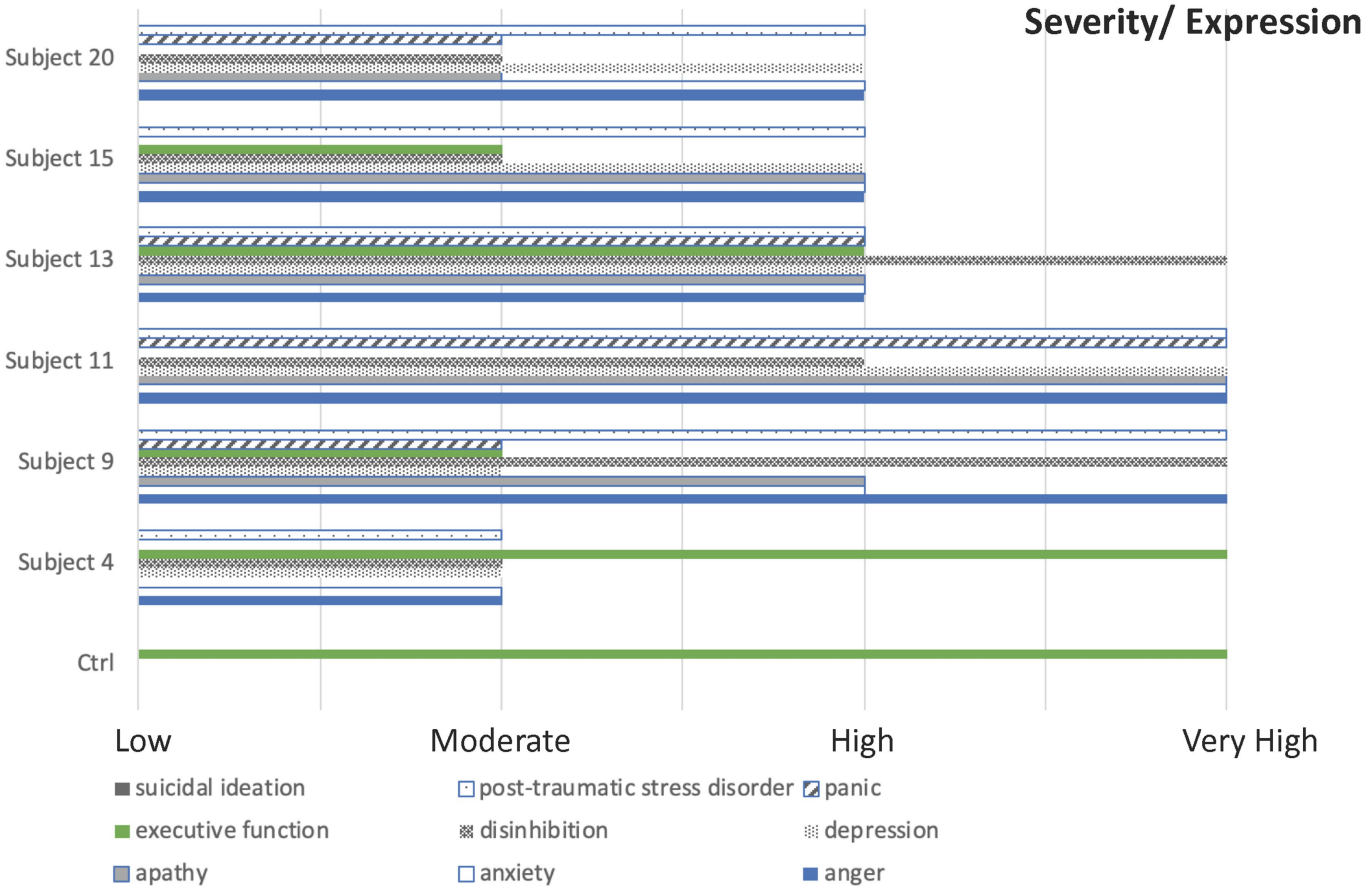
A qualitative neuropsychological description. A description of *N* = 6 subjects with indications of Havana Syndrome described in terms of 9 neuropsychological constructs where severity is scaled onto a discrete qualitative scale from 0 or minimal to 3 or most severe. The reference control condition was defined as a stable resting state with minimal severity and maximum function.

To test the hypothesis that HS resulted from a characteristic dysregulation of neurotransmission, neuro-inflammatory response, we compared the neuropsychological profiles predicted by our mechanistically informed network model to those reported in a subset of *N* = 6 individuals who underwent detailed neuropsychological testing (5). We focused on this particular subset of individuals for several reasons. First, since the case definition is still evolving, we expected significant heterogeneity across subjects assigned to a HS phenotypic group. Secondly, this variability is compounded by the fact that the exposure source or sources are unknown, and the extent of exposure is almost certainly not uniform from one individual to the next. As a result of these uncertainties, in group definition and variability in the extent of illness, we argue that it is more appropriate to assess this heterogeneity outright by preserving resolution at the level of the individual rather than attempting to explain an average neuropsychological profile thought to be descriptive of the group as a whole. Accordingly, we used functional measures reported by Swanson et al. (5) at the level of the individual to test our network model. These included assessments of the Beck Depression Scale (36), the Beck Anxiety Inventory-Revised (37–39), and the Post-Traumatic Stress Disorder Checklist-5 (40–42). In addition, we used assessments of the subscales that constitute the Brief Mood Survey, namely Depression, Suicidal Urges, Anxiety, Panic, and Anger. Finally, we used data describing function in individual components of the Frontal Systems Behavior Scale (43, 44) specifically Apathy, Disinhibition and Executive Dysfunction. As mentioned in the previous section, all numerical scores were translated using a simple range normalization scheme into 4 qualitative levels of severity ranging from Low (0) to Very Severe/Very High (3) in support of the discrete qualitative logic used to describe the progression in time of the network from one neurobehavioral expression profile to the next. The resulting qualitative expression scores for each of the 9 neuropsychological measures are illustrated in Figure 3 for each subject and reported in Supplementary Table S3. As Executive Dysfunction was not reported explicitly in the Knowledge Graph database (Elsevier, Amsterdam) (21), we reversed the scale and used the opposite construct of executive function in the network model with low executive function being substituted for high executive dysfunction. These reported neuropsychological profiles are being used in this analysis to validate a theoretical model instead of the more conventional approach of constructing a naïve model *de novo* by extracting patterns directly from the data. As a result, such a hypothesis-driven approach is especially well-suited for the analysis of small sample sizes and unobserved variables (e.g., the neurologic markers in this network).

## 3. Results

### 3.1. HS through the lens of mTBI neurological pathways

As an extension of an earlier model capturing neurotransmission and neuroinflammatory processes relevant to mTBI, the current network consists of 29 markers of brain signaling, 9 neuropsychological markers and an exogenous stressor. These 39 nodes are linked through 273 regulatory interactions supported by 9,032 peer-reviewed publications (Figure 1). This corresponds to a connection density of roughly 18% consistent with estimates of neuroanatomical connectivity reported in models of Macaque brain enriched for fundamental structural and functional motifs (25, 45). The neurotransmitter substance P emerged as a key information broker in the network with the highest betweenness centrality of all nodes since it is a key first responder to many stressors with involvement in pain perception, neuroinflammation, mood and cognition (Supplementary Table S2). Substance P also recruited the largest number of downstream nodes giving it the highest closeness centrality and supporting a high degree of involvement in the broader network.

This body of over 9,000 peer-reviewed publications translates into a median support of 9 citations per interaction with the reciprocal relationship between anxiety and the stress hormone CRH being documented in over 400 publications. Moreover, 162 (approximately 60%) of the text mined interactions were documented in 5 or more publications with 19 interactions being supported by over 125 citations each (Supplementary Figure S1). Conversely, 38 of these 273 relationships were supported by only 1 publication. These were flagged as low confidence relationships and their role in the network tested against the data. In the context of this network and the available neuropsychological profiles, the retention in the model of all 38 low confidence relationships offered the best alignment of model predictions and experimental observations. Thus, a set of parameter values defining a bio-behavioral regulatory program were identified that supported agreement of model prediction with an overall departure of 6% aggregated across all 9 neuropsychological constructs and the 6 subjects. Significantly, neuropsychological profiles in four out of the six cases were captured with less than 5% disagreement (Manhattan distance) with two of these being recovered exactly (subjects 4 and 15). The largest departures from predicted network behavioral profiles coincided with the more severe symptom profiles, namely subjects 9 and 11, with the latter being the least well explained by documented mTBI response mechanisms alone. Although elevated symptom burden was predicted in both cases, the severity fell below that exhibited by the subjects in question. This was especially true of the Brief Mood Survey anger scores and the PTSD symptom burden scores suggesting the involvement of illness mechanisms beyond those associated with classic mTBI symptomatology.

### 3.2. Characteristic neurological marker co-expression

**Figure 4:**
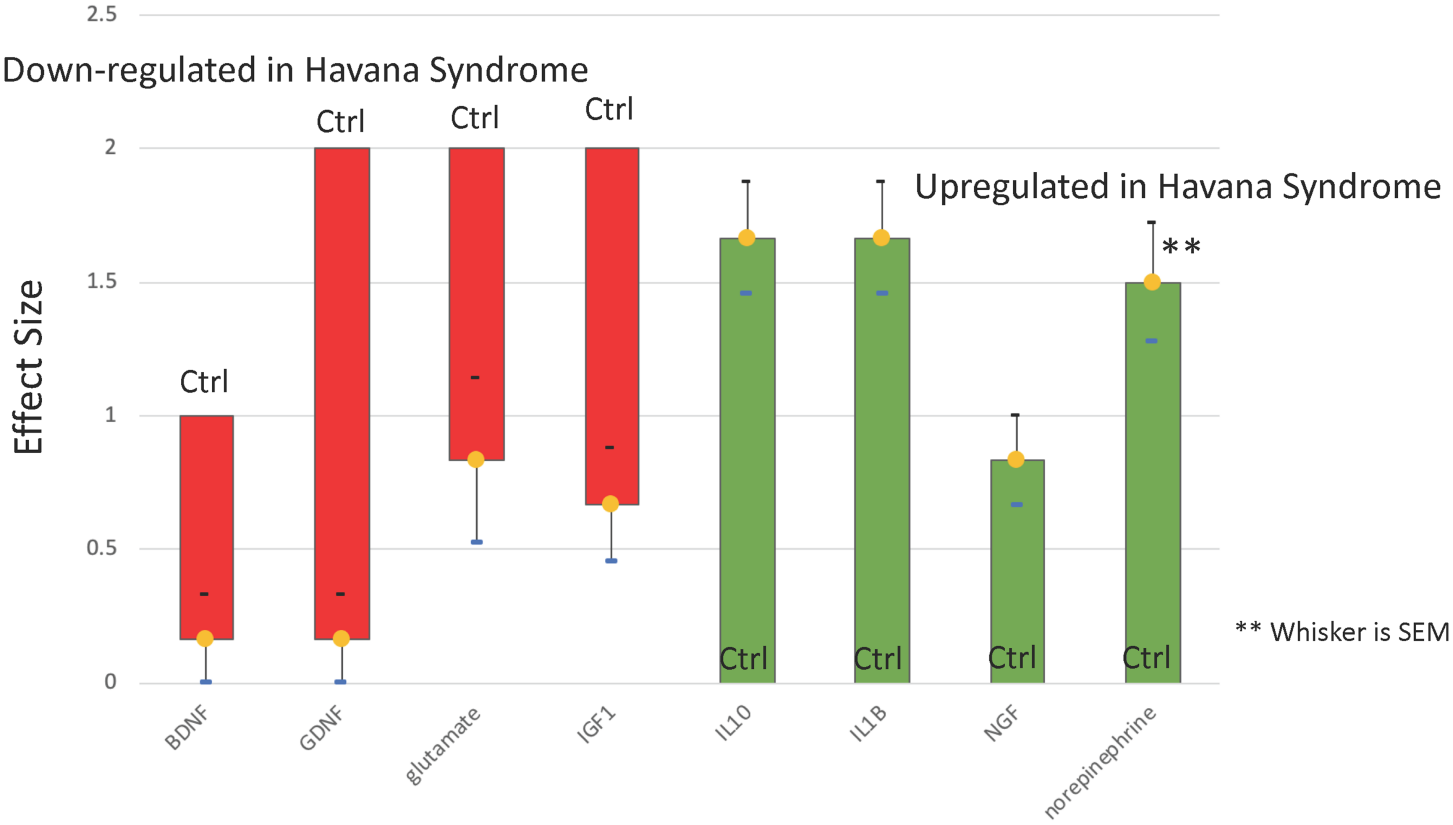
Predicted dysregulation of neurological biomarkers. Predictions based on a regulatory network model of known neurotransmission and neuro-inflammatory pathways indicate that 8 of 29 neurological markers might be differentially expressed at FDR ≤ 5% in the group of *N* = 6 Havana Syndrome subjects compared to a minimum severity control reference profile (Ctrl). The model-based co-expression profile characteristic of Havana Syndrome would consist of depressed neurotrophic factors BDNF, GDNF, IGF1, and glutamate in the context of over-expression of IL-10, IL-1B, NGF, and norepinephrine.

It is credible to predict expression patterns of unobserved neurotransmitters and neuroinflammatory markers capable of driving specific neuropsychological profiles given the strict interdependency between bio-behavioral mediators imposed by the structure of the regulatory network. Profiles for these unmeasured biomarkers were derived in concert with the parameter values for the regulatory logic such that these predicted expression profiles for each subject optimally satisfied the network structure, its regulatory program, and best supported the alignment of the predicted neuropsychological profiles observed. These biomarker expression profiles are presented for each of the 6 subjects in Supplementary Table S4. Although we expected substantial variability, HS subject profiles were compared as a group (*N* = 6) to the reference profile predicted for the idealized control (*N* = 1) corresponding to minimal symptom burden and maximal executive function. This was performed by using a one-sample *t*-test with a Benjamini Hochberg correction for multiple comparison across all 29 markers. Results suggested significant upregulation of IL-1B, IL-10, NGF, and norepinephrine in the HS group, as well as the downregulation of BDNF, GDNF, IGF1, and glutamate (FDR < 5%). Slight elevations in CRH and IL-6 were also predicted unanimously across all HS subjects (Figure 4). Interestingly, though substance P occupies a position in the network whereby it may act as a key information broker, it was not predicted to be differentially expressed and would appear to be equally active in this role in either group.

### 3.3. Persistence and progression

**Figure 5:**
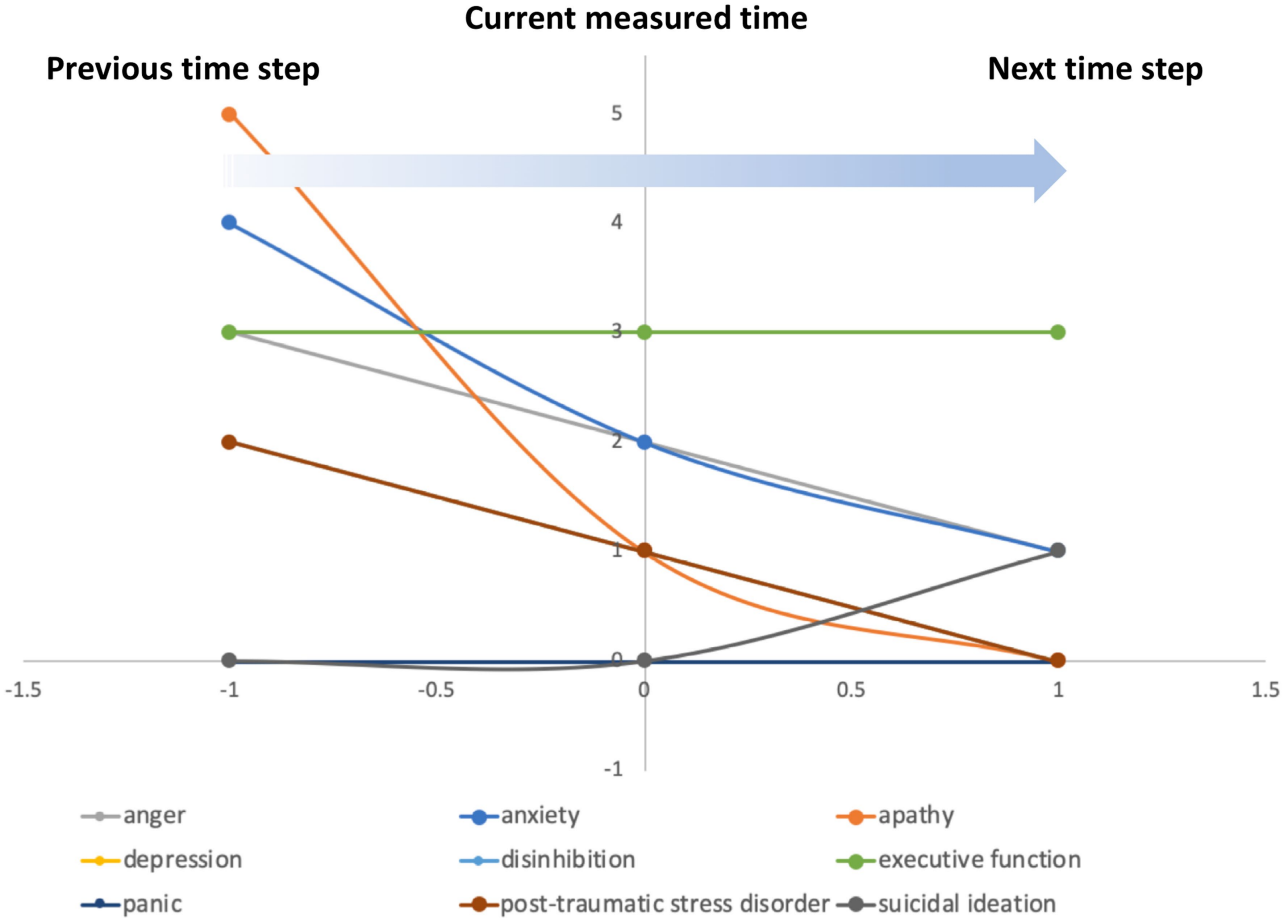
Predicting the current course of illness. The decisional logic rules that allow the regulatory network to support the symptom severity and functional profiles observed in the *N* = 6 subjects implicitly determine the profiles that must have preceded the current cross-sectional observation as well as the next state to which the network is expected to progress. As in subject 4 (above), severity scores decrease monotonically, with exception of suicidal ideation score and apathy, with function improving or remaining high suggesting a trend towards a slow partial recovery in this group.

In concert with the network structure, the decisional logic governing the flow of information across the network will dictate the expression profiles available to precede the cross-sectional observation as well as the most logical next state. If this next logical state is exactly the same as the current state, then a dynamically stable persistent state has been reached. Such a persistent end state can be used to represent chronic pathology, or one that is galvanized by homeostatic regulation. The likelihood of these 6 neuropsychological profiles representing a persistent pathology was tested by directing the parameter search to identify decisional logic programs that when applied to this regulatory network circuit would converge to these symptom profiles and remain at steady state. When this constraint was applied, no feasible parameter sets were found that could govern the behavior of the network. This suggests that the 6 neuropsychological profiles in question were not dynamically stable end points, but instead represented cross-sectional snapshots recorded along the course of a still active response trajectory. Indeed, the predicted progression suggests a slow recovery trend even for the most severe case. The trajectory predicted for subject 4 (Figure 5) is representative of all 6 subjects where executive function remained elevated while indicators of symptom burden were predicted to adhere to a slow recovery trend. At the level of neurologic mediators, this is accompanied by a somewhat consistent trend across the group towards recovery in expression of neurotrophic factors like BDNF. This is further supported by continued stimulation of repair through increased expression of growth factors like NGF and tumor suppressor NF1, accompanied by decreasing activation of stress responsive CRH and NPY with concurrent decreasing expression of neuroinflammatory markers IL-6 and TNF (Supplementary Table S5).

### 3.4. A NLP-driven exploration of potential initiating exposures

The probable signature characteristic of this small group of subjects suspected of suffering from a variant of HS consisted of the differential expression of 8 neurotransmitters and neuroinflammatory markers so we conducted a focused text-mining of the literature in an attempt to extract potential exposure sources known to alter these markers. The MedScan NLP engine made available through the Elsevier Text Miner interface (Elsevier, Amsterdam, NL) was used to extract such documented relationships from the broader family of electromagnetic exposures, and where these upregulated or downregulated each of the 8 differentially expressed biomarkers. Given that the symptoms reported in presumed HS cases by Swanson et al. (5) did not appear consistent with the effects of exposure to ionizing and thermal radiation, these were excluded from the search. This also redirected the search away from the substantial body of literature describing the effects of ionizing radiation that are delivered therapeutically (e.g., in interventional radiation oncology). Results of this focused text mining showed that the characteristic down or upregulation of almost all predicted HS markers could potentially be caused by pulsed low frequency electromagnetic fields (EMF) including radio frequency radiation (Supplementary Table S6). Of the changes predicted in these 8 biomarkers of interest, only the downregulation of GDNF and IGF1 undeniably required the inclusion of ionizing radiation as a source.

## 4. Discussion

The current hypothesis-driven analysis was conducted to determine if and to what extent neuropsychological profiles reported in a limited number of subjects satisfying the case definition of Havana Syndrome might be explained by dysregulation of established bio-behavioral pathways associated with mTBI. The degree of alignment between regulatory network predictions and the observed symptom profiles would suggest HS may have very real neurological underpinnings and that these may overlap significantly with mechanisms involved in mTBI in at least a segment of these individuals. That said, the severity of the symptom profiles in at least 2 of the 6 cases considered here was under-represented by this model of mTBI. This would suggest that while there might exist a strong commonality with mTBI, HS may also impact and recruit the involvement of additional neurokinetic mechanisms unique to this condition. Indeed, deviation from this reference dysregulation in mTBI may be an important contributor to phenotypic stratification and corresponding clinical intervention in individuals with this unique variant of classical mTBI. With regard to the progression of illness, the dynamic behavior predicted by the model regulatory network did not support the persistence of the symptom profiles observed, suggesting instead that these subjects were engaged in a slow recovery process, even for the most severe case. In other words, these individuals were not expected to be bound in a regulatory trap that might be refractory to intervention. This is consistent with observations of Swanson et al. (5) indicating that these individuals in their care appeared responsive to rehabilitation. It should be noted however that a diagnosis of AHI as mTBI may be a misnomer. AHI patients diagnosed with mTBI (as well as their families and employers) may believe that since it is termed “mild” there should be rapid improvement of symptoms. Yet, we know that approximately 5% of Veterans diagnosed with mTBI have continued symptoms for months to years after the experiencing event (blast, blunt trauma, and whiplash injury, etc…) (46). Lastly, clinicians and patients must be educated on stigma related to reporting symptoms and receiving treatment. This is similar to the stigma associated with behavioral health treatment, and it is our opinion that many patients may not present due to fear that it will impact their careers.

It has been proposed that the HS inciting multisensory sensory phenomena may stem from exposure to directed, pulsed radiofrequency energy (3) extending into microwave frequencies (47). There have been some efforts in the literature to explain the mechanism by which pulsed radiofrequency/ microwave (RF/MW) radiation could cause brain injury. At the level of neurologic markers, predicted expression levels in these 6 subjects suggested a coordinated upregulation of IL-1B, IL-10, NGF, and norepinephrine with concurrent downregulation of BDNF, GDNF, IGF1, and glutamate. The biological effects of exposure to electromagnetic fields have been previously reported in animal and human studies. For instance, Santini et al. (48) found that low frequency electromagnetic fields can adversely affect immune system and neural health among humans. Specifically, Hosseinabadi et al. (49) found that IL-1B, a potent pro-inflammatory cytokine, was significantly elevated in a group of individuals exposed to low frequency electromagnetic fields, compared to the unexposed group. Further, a subgroup analyses (49) by the latter showed that within the exposed group, subjects with the highest levels of exposure also expressed higher levels of IL-1B compared to the other less exposed subgroups. Increased IL-1B is also a key mediator in immune response to infection and injury, and associated with autoinflammatory diseases (50). Increased presence of IL-1B is also seen in Alzheimer’s disease (51) as well as in mild cognitive impairment (52). Accordingly, predictions of elevated IL-1β in the current study may point to a potential role of pulse repetition frequency (PRF) energy in HS. Likewise, the elevated IL-10 predicted by the current model is consistent with the literature documenting the immunomodulatory influences of pulsed electromagnetic fields (PEMF) and pulsed radiofrequency energy (PRFE). For instance, a rodent study that used therapeutic exposure to PEMF or PRFE found significant elevation of IL-10 (53, 54). Further, IL-10 activation as a result of extremely low-frequency magnetic field exposure is also documented (55). As IL-10 has an inhibitory effect on IL-1B (56, 57), PEMFs are generally found to decrease IL-1B (58). While counterintuitive at first glance, the concurrent overexpression of IL-1B and IL-10 predicted by the current model could serve as further evidence that these cross-sectional observations are indeed still very much a part of an ongoing dynamic response to injury (59) and do not represent a resting state equilibrium.

Stimulated by IL-1B, the release of NGF, a neurotrophic protein, is essential for normal growth and differentiation of sensory and sympathetic neurons as well as for nerve regeneration and neuroplasticity (60). There is some evidence that PEMF is associated with increased NGF (61). Moreover, the neuromodulation of extremely-low-frequency magnetic fields on a rat sample varied across the age of the animals, such that young rodents expressed higher NGF, compared to older rodents (62). Similarly, norepinephrine, a monoamine molecule, is primarily involved in autonomic nervous system and also has a crucial role in brain development and health (63). In a review of the literature, it is reported that sub-acute electromagnetic radiation increased norepinephrine and triggered a cascading effect on other catecholamines such as epinephrine (64). Similarly, studies on rodents found that low-intensity magnetic field increased muscle norepinephrine (65) and norepinephrine in the pineal glands (66). Notably, Aboul Ezz et al. (67) reported a significant elevation of norepinephrine among rats which were exposed to electromagnetic radiation daily for 4 months which subsequently decreased when the exposure was stopped.

In this work we also predict the transient down-regulation of various neurotrophic and growth factors including BDNF, GDNF, IGF1, and the essential neurotransmitter glutamate. Brain-derived neurotrophic factor (BDNF) belongs to the family of neurotrophins, and is crucial to brain function, particularly memory (68), and is key to neuroprotection (69). Consistent with the findings of this study, Tian et al. (70) reported that rats exposed to electromagnetic pulse showed decreased BDNF compared to a control group. Because increased BDNF is essential to neurogenesis and brain health, the downregulation of BDNF is a risk factor for various neuropsychiatric diseases including Alzheimer’s disease and stroke as well as various metabolic disorders (68). Similarly, another neurotrophic factor, GDNF is especially important in protecting auditory neurons against auditory assault and trauma (71). Therefore, PEMF-induced concomitant downregulation of BDNF and GDNF may be especially relevant to HS. Likewise, insulin-like growth factor 1 (IGF-1) is the most abundant growth factor in the bone matrix that is responsible to maintain bone mass (72). Sato et al. (73) examined the effect of electrical stimulation on the production of IGF-1 protein and found that 24 h after applying 10 mA (measured in milliamps (mA)), IGF-1 decreased by nearly 40% compared to IGF-1 proteins which were exposed to 0 mA electrical stimulation. Further, glutamate, a major excitatory neurotransmitter with a crucial role in cognition, movement, learning, and memory (74) was also reported to be downregulated in young adult rats exposed to electromagnetic radiation for 1 month (75). It should be noted, however, that there is a large section of the literature indicating the upregulation of IGF-1 (76, 77) and glutamate (55) in response to PEMFs.

Overall, this exploratory simulation study offers predictions consistent with a limited body of literature on the effects of PEMF exposures suggesting that the latter may indeed exert changes in neurochemistry and neural responses. Importantly however, the findings of the current study should be interpreted with caution as these effects are also highly time-dependent and differ significantly based on the dose, duration of exposure, and the source of EM frequency (78). In addition, it must be emphasized that the text-mining for potential exposure sources was conducted in a bottom-up fashion. In other words, the Elsevier ontology and automated reader MedScan were used to search the Elsevier corpus for statements describing the focused modulation by a specific source of those molecular biomarkers predicted to be differentially expressed by the model. This focus on molecular effects rather than clinical outcomes or symptoms greatly reduced the applicable body of literature to one consisting for the most part of animal studies. As a result, a broader search directed at alterations in the neuropsychological profiles may point to additional plausible sources of exposure, for example infrasonic exposure (79).

Certainly our ability to accurately extract information from text continues to evolve at a rapid pace as do the ontologies upon which these tools rely. As a result, the text mining conducted here by no means delivers an exhaustive representation of our current knowledge. Indeed, though the trends were represented, the departure of model predictions from the symptom profiles observed in the more severely affected subjects would suggest that some important regulators may still be absent from this first literature-based model (e.g., VEGF, FGF, and others). Our group currently working to integrate additional automated readers and ontologies as well as pathway schema in the creation of these network models using environments such as INDRA (80). Using tools such as this a formal gap analysis would be warranted as larger data sets become available. Similarly, while the experimental data was used to validate a mechanistic model rather than create such a model *de novo*, the basic assumption in the interpretation of the results remains that the 6 subjects profiled here are representative of the broader group of subjects reporting AHI and what is still an evolving definition of HS. Only further validation in a larger sample population can motivate the inclusion of additional molecular mediators as well as confirm or dispel similarities shared by AHI subjects by studying both healthy control and additional illness (e.g., PTSD and mTBI) groups. Finally, specificity of signatures to given exposure-related phenotypes would benefit from a broader description of symptom burden and clinical presentation. Nonetheless, we propose that the current study offers an analytical framework that is rooted in established knowledge of basic physiological pathways offering a mechanistically governed assessment of data. Such a hypothesis-driven approach rooted in prior knowledge is directed at explaining the data instead of simple prediction. The review of exposure sources known to affect the altered expression of molecular mediators predicted by the model and the divergent effects of PEMFs on neural health according to dose, frequency and duration of exposure (78) may possibly explain at least in part the heterogenous symptomatology in subjects with suspected HS. As “the brain is the battlefield of the future (81)”, this paper may serve in drawing the attention of clinicians to AHIs, and the emerging threat of directed energy weapons capable of rendering victims (military and non-military) incapable of function without inflicting visible injury. Additionally, results of this initial analysis highlight the potential significant overlap of mTBI-relevant regulatory physiology and the opportunity of redirecting mTBI treatment protocols to guide in the treatment of this complex condition.

## Data availability statement

The original contributions presented in the study are included in the article/Supplementary material, further inquiries can be directed to the corresponding author.

## Ethics statement

This retrospective study was approved by the institutional review board of the University of Pennsylvania’s Perelman School of Medicine. Written informed consent for participation was not required from the participants or the participants’ legal guardians/next of kin in accordance with the national legislation and institutional requirements. This is an automated text-mining of peer reviewed published results in the Elsevier Corpus – each of which was approved by their respective IRB as a condition of publication. The study was conducted in accordance with the local legislation and institutional requirements.

## Author contributions

TC was primarily responsible for literature review, model creation, results analysis, and manuscript writing. MM and JP were primarily responsible for network analysis, and generation and interpretation of results. JT was primarily responsible for supervision of manuscript writing, expertise on computational modeling techniques, and clinical applications. MR, RB, MC, RF, and JB contributed to the design of the study as well as the interpretation of results, assessment of clinical validity, and writing of the manuscript. GB was responsible for oversight and funding of project, definition and supervision of the research, manuscript writing, and expertise on computation modeling techniques. All authors contributed to the article and approved the submitted version.

## Footnotes

^1^ https://cytoscape.org

^2^ https://www.python.org/

## Author disclaimer

The opinions and assertions contained herein are the private views of the authors and are not to be construed as official or as reflecting the views of the US Department of Veterans Affairs, the US Department of Defense, Rochester Regional Health, or Elsevier BV. Pathway Studio (© 2020), Elsevier Text Mining and Elsevier Knowledge Graph are trademarks of Elsevier Limited. Copyright Elsevier Limited except certain content provided by third parties.
