# Supplementary material for: A regulatory pathway model of neuropsychological disruption in Havana syndrome
Source: Front Psychiatry. 2023 Oct 27;14:1180929. doi: 10.3389/fpsyt.2023.1180929 (PMC10642174; doi:10.3389/fpsyt.2023.1180929)
Supplement: Supplementary file 1 [file Image_1.pdf]

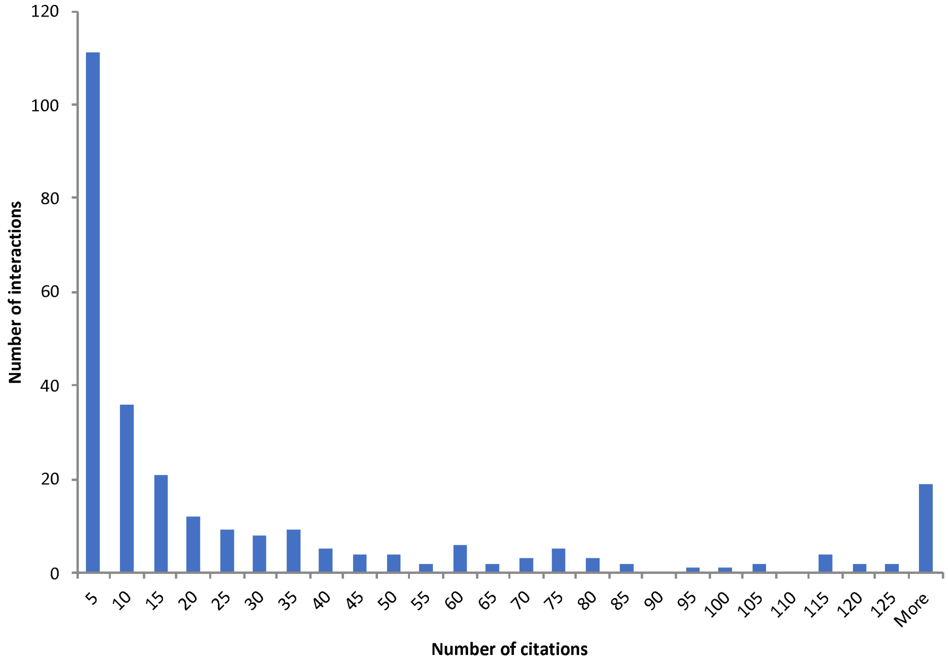

Supplementary Figure S1. *Literature support of regulatory interactions*. In a network consisting of 39 nodes linked by 273 interactions, more than half (162 interactions) were supported by 5 citations or more, with close to 20 being supported by over 125 publications.
